# Maternal mRNA deadenylation is defective in in vitro matured mouse and human oocytes

**DOI:** 10.1038/s41467-024-49695-y

**Published:** 2024-07-02

**Authors:** Yusheng Liu, Wenrong Tao, Shuang Wu, Yiwei Zhang, Hu Nie, Zhenzhen Hou, Jingye Zhang, Zhen Yang, Zi-Jiang Chen, Jiaqiang Wang, Falong Lu, Keliang Wu

**Affiliations:** 1 College of Life Science, Northeast Forestry University, Harbin, 150040 China; 2 State Key Laboratory of Molecular Developmental Biology, Institute of Genetics and Developmental Biology, Chinese Academy of Sciences, Beijing, 100101 China; 3 State Key Laboratory of Reproductive Medicine and Offspring Health, Center for Reproductive Medicine, Institute of Women, Children and Reproductive Health, Shandong University, Jinan, 250012 China; 4 College of Life Science, Northeast Agricultural University, Harbin, 150030 China; 5 University of Chinese Academy of Sciences, Beijing, 100049 China; 6 Research Unit of Gametogenesis and Health of ART-Offspring, Chinese Academy of Medical Sciences (No. 2021RU001), Jinan, Shandong 250012 China

**Keywords:** Embryology, Oogenesis, RNA decay, Transcriptomics

## Abstract

Oocyte in vitro maturation is a technique in assisted reproductive technology. Thousands of genes show abnormally high expression in in vitro maturated metaphase II (MII) oocytes compared to those matured in vivo in bovines, mice, and humans. The mechanisms underlying this phenomenon are poorly understood. Here, we use poly(A) inclusive RNA isoform sequencing (PAIso-seq) for profiling the transcriptome-wide poly(A) tails in both in vivo and in vitro matured mouse and human oocytes. Our results demonstrate that the observed increase in maternal mRNA abundance is caused by impaired deadenylation in in vitro MII oocytes. Moreover, the cytoplasmic polyadenylation of dormant *Btg4* and *Cnot7* mRNAs, which encode key components of deadenylation machinery, is impaired in in vitro MII oocytes, contributing to reduced translation of these deadenylase machinery components and subsequently impaired global maternal mRNA deadenylation. Our findings highlight impaired maternal mRNA deadenylation as a distinct molecular defect in in vitro MII oocytes.

## Abstract (web summary)

The study reveals defective maternal mRNA deadenylation in oocytes matured in culture dishes versus natural ones, potentially explaining lower fertility in assisted reproduction with immature oocytes.

## Introduction

Female infertility is becoming an exacerbating reproductive problem world wide, for which the assisted reproductive technology (ART) is an effective treatment^1^. In vitro maturated metaphase II (in vitro MII) oocytes were introduced as an assisted reproductive technology for patients with polycystic ovarian syndrome (PCOS)^2^ that affects a large population globally^3^ and patients with severe ovarian hyperstimulation syndrome (OHSS) during previous in vitro fertilization (IVF) treatments^4^. Currently, in vitro MII oocytes can be adopted as an option in almost all areas of fertility clinics, including PCO-like ovaries, resistant ovary syndrome, previous failed IVF attempts, and oocyte maturation problems^5^. Additionally, in vitro MII oocytes can be useful for preserving fertility in situations such as emergency oocyte retrieval due to malignancies^6^. A baby from donor in vitro MII oocytes was born in 1991^7^, and a baby from the mother’s own in vitro MII oocytes was born in 1994^8^. As such, in recent years, in vitro MII oocytes have gained increasing attention for their feasibility, safety, reproducibility, cost-effectiveness, and lack of OHSS risk^8,9^.

Previous studies have demonstrated that embryos derived from in vitro MII oocytes have lower success rates of preimplantation development, pregnancy, and birth than those derived from mature in vivo MII (in vivo MII) oocytes^10^. The nuclear and cytoplasmic maturation in in vitro MII oocytes determines the performance of their oocytes, the quality of the embryo, and clinical outcomes^5,11^. The nuclear maturation can be evaluated with a microscope based on the first polar body extrusion, and possibly the dynamic changes of chromatin states^12^. The cytoplasmic maturation defects during the in vitro maturation process include altered spindle positioning, mitochondrial membrane potential, the number of endoplasmic reticulum clusters, and the cortical actin cytoskeleton thickness in in vitro MII oocytes compared with in vivo MII oocytes^13,14^. Global gene expression profiling using human whole-genome arrays provides compelling evidence for the relative developmental incompetence of in vitro MII oocytes^15^, demonstrating that over 2000 genes were expressed at twofold or greater levels in in vitro MII oocytes compared with in vivo MII oocytes. In vitro MII oocytes in mice and bovines were also demonstrated to be of elevated expression patterns^16,17^. Nonetheless, the mechanisms underlying cytoplasmic maturation defects and upregulated gene expression profiles in in vitro MII oocytes remain largely unknown.

As transcription is silent, post-transcriptional regulation of maternal mRNA plays a dominant role in oocyte maturation, particularly deadenylation-dependent maternal mRNA decay. The CCR4-NOT deadenylase and its adapter *Btg4* are critical regulators for this process^18–20^. In addition, *Cnot7* and *Cnot6l*, which encode catalytic subunits of the CCR4-NOT deadenylase, and *Btg4* are dormant maternal mRNAs that needs to be translationally activated through cytoplasmic polyadenylation during mouse oocyte maturation^18,19^. We demonstrated that maternal mRNAs are subjected to deadenylation-dependent decay during oocyte maturation in mice, rats, pigs, and humans^21–25^. Therefore, we hypothesize that the global gene upregulation observed in the in vitro MII oocytes is caused by impaired global deadenylation.

In this study, we employed poly(A) inclusive RNA isoform sequencing (PAIso-seq), a method that enables the measurement of full-length transcriptomes with complete poly(A) tails from a single oocyte^26^, to sequence the transcriptome-wide poly(A) tails in MII oocytes matured in vivo or in vivo in both mice and humans. By analyzing the transcriptome-wide poly(A) tail length distribution of maternal mRNA in in vitro and in vivo MII oocytes, we found that the poly(A) tail deadenylation was compromised in in vitro MII oocytes. Additionally, we observed a malfunction in the cytoplasmic polyadenylation of *Btg4* and *Cnot7* mRNAs in in vitro MII oocytes of both mice and humans, leading to a reduction in the protein levels of Btg4 and Cnot7, as confirmed in mice. Therefore, our findings highlight that the impaired deadenylation of maternal mRNA represents a critical defect in in vitro MII oocytes in both mice and humans, and pave the way for improving the quality of in vitro MII oocytes in assisted reproductive technologies.

## Results

### Abnormal maternal gene expression in in vitro MII oocytes in mice and humans

**Fig. 1:**
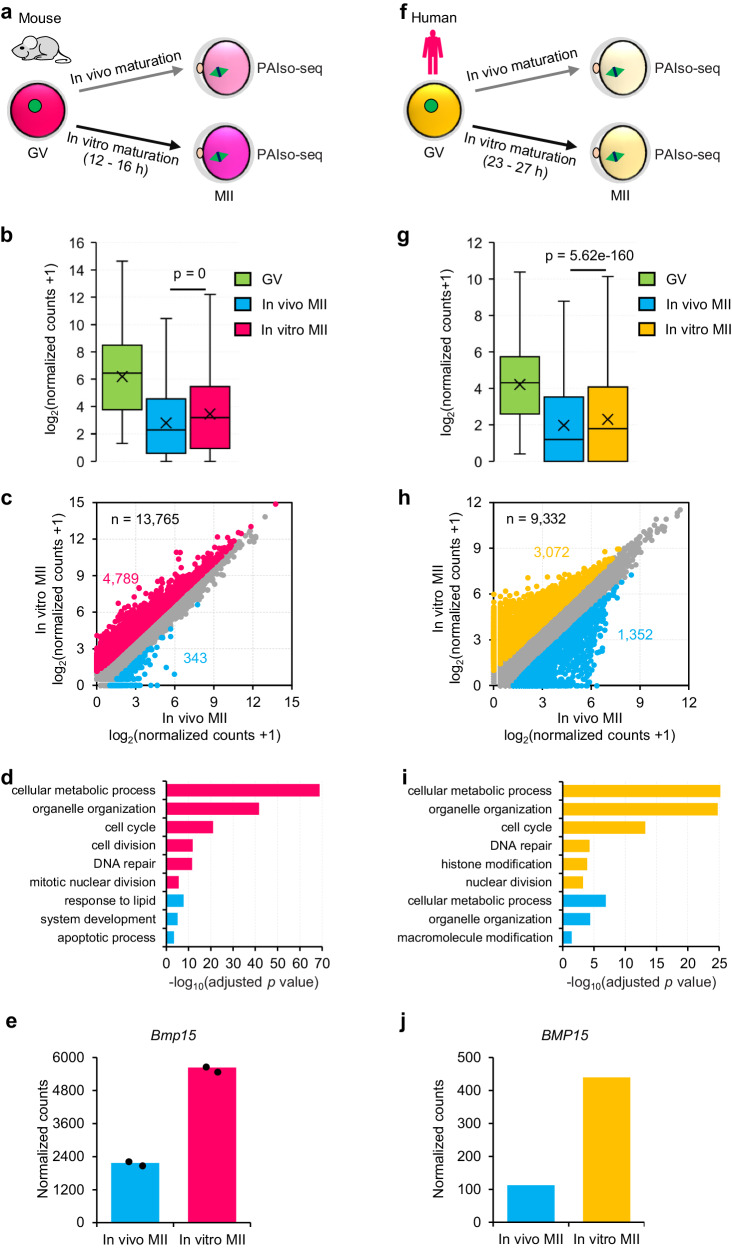
Abnormal maternal gene expression in mouse and human oocytes matured in vitro. **a**, **f** Illustration of the in vitro and in vivo oocyte maturation experiments in mouse (**a**) and human (**f**) oocytes. **b**, **g** Box plots for the normalized counts (in log2 scale) of genes (**b**, *n* = 14131; **g**, *n* = 12013) in germ-vesicle (GV) and in vivo maturated metaphase II (in vivo MII) or in vitro maturated metaphase II (in vitro MII) oocytes in mice (**b**) or humans (**g**). **c**, **h** Scatter plot for the normalized counts of individual genes in MII oocytes matured in vivo or in vitro in mice (**c**) or humans (**h**). Each dot represents one gene. Genes with at least 1 read in one of the samples are included in the analysis. The number of genes included in the analysis and the number of differentially expressed genes are shown on the graphs. Genes upregulated in the in vitro MII oocytes are in red (**c**) or orange (**h**), while those downregulated in the in vitro MII oocytes are in blue. The differential expression is defined by a twofold cutoff. **d**, **i** Gene Ontology (GO) analysis of genes upregulated (red in **d** and orange in **i**) or downregulated (blue) in the in vitro MII oocytes compared to in vivo MII oocytes in mice (**d**) or humans (**i**). The GO analysis and its associated statistical results were performed with the g:Profiler tool. **e**, **j** Normalized counts of *Bmp15* in the in vitro and in vivo MII oocytes for two biological replicates of mice (**e**) or combined data of humans (**j**). Individual data points are shown on the plots. All *p* values are calculated by a two-tailed Student’s *t-*test. For all the box plots, the “×” indicates the mean value, the central line represents the median value, the top and bottom of the box represent the value of the 25th and 75th percentile, and the boundary of the lower and the upper whiskers represent the minimum and the maximum value of the data set, respectively. The read counts are normalized by the counts of reads mapped to protein-coding genes in the mitochondria genome, if normalization is indicated. Source data are provided as a Source Data file.

The deadenylation and cytoplasmic polyadenylation of the poly(A) tail of maternal mRNA both play essential roles during oocyte maturation^27,28^. Therefore, we performed PAIso-seq^26^ to analyze the poly(A) tail inclusive transcriptome in in vitro and in vivo MII oocytes as well as the germinal vesicle (GV) oocytes and obtained PAIso-seq data of good quality in both mice and humans (Fig. 1a, f and Supplementary Fig. 1). Global maternal mRNA decay through poly(A) tail deadenylation takes place during oocyte maturation^18–20^. Therefore, it is not suitable to normalize transcriptome using a regular method based on the total number of sequenced reads. Mitochondrial genome-encoded (MT-encoded) polyadenylated transcripts have been proven to be a good internal reference for the normalization of the transcriptome from cells depleted of CNOT7, CNOT8, PAN2, PAN3, and PARN^29^, because the MT-encoded polyadenylated transcripts are located in mitochondria which are not affected by the cytoplasmic poly(A) tail regulator^29^. Consistently, in both human and mouse GV, in vitro matured MII, as well as in vivo matured MII oocytes, the poly(A) tails of MT-encoded polyadenylated mRNAs are minimally affected (Supplementary Fig. 2). Therefore, we employed MT-encoded polyadenylated mRNAs for the normalization. We found that the individual mRNA abundance decreased significantly during maturation in both mice and humans (Fig. 1b, g). This is consistent with the well-known global reduction of maternal mRNA during mammalian oocyte maturation, as measured by RNA-seq or microarray^15,18,19^. The transcriptional level of many individual genes was significantly higher in in vitro than in in vivo MII oocytes in mice and humans (Fig. 1b, g), indicating that global maternal mRNA decay is impaired in in vitro MII oocytes. We found that 4,789 genes increased, while only 343 genes decreased in in vitro compared to in vivo MII oocytes in mice (Fig. 1c and Supplementary Data 1), and 3072 genes increased while only 1352 genes decreased in in vitro compared to in vivo MII oocytes in humans (Fig. 1h and Supplementary Data 2). Statistical tests were not conducted here due to the lack of replicates for human samples. However, the conclusion is the same that several thousand genes increased with several hundred or tens of genes decreased for mouse samples if differential gene expression was statistically tested with edgeR or student’s *t*-test (Supplementary Data 1). The upregulated genes enrich in cellular metabolic process, organelle organization, cell cycle, as well as DNA repair in both mice and humans (Fig. 1d, i). This indicates the presence of conserved defects in in vitro MII oocytes between mice and humans. *Bmp15* is known to inhibit follicle maturation and is downregulated during this process^30^; we found that it is upregulated in in vitro compared to in vivo MII oocytes in both mice and humans (Fig. 1e, j). Our findings are consistent with previous global gene expression profiling comparing in vitro and in vivo MII oocytes in humans and mice^15,16^ and were similar when comparing pluripotent stem cells (PSCs)-derived MII oocytes and in vivo MII oocytes of mouse^31^. Together, these results indicate that the impaired maternal mRNA decay is one conserved defect in in vitro MII oocytes in both mice and humans.

### Impaired maternal mRNA deadenylation in in vitro MII oocytes of mouse and human

**Fig. 2:**
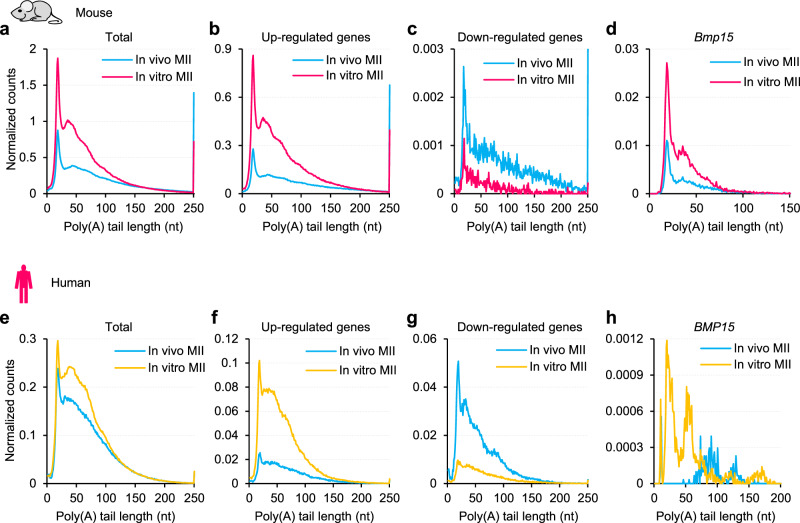
mRNA deadenylation is impaired in mouse and human oocytes matured in vitro. Histogram of poly(A) tails length of all transcripts (**a**, **e**), transcripts of the in vitro MII oocyte-upregulated genes (**b** red genes (*n* = 4789) in Fig. 1c; **f** orange genes (*n* = 3072) in Fig. 1h), transcripts of the in vitro MII oocyte-downregulated genes (**c** blue genes (*n* = 343) in Fig. 1c; **g** blue genes (*n* = 1352) in Fig. 1h), or transcripts of *Bmp15* in in vivo or in vitro MII oocytes in mice (**a**–**d**) or humans (**e**–**h**). Histograms (bin size = 1 nt) are normalized by counts of reads mapped to protein-coding genes in the mitochondria genome. Transcripts with poly(A) tails of at least 1 nt are included in the analysis. Transcripts with poly(A) tail lengths greater than 250 nt (150 nt for **d** and 200 nt for **h**) are included in the 250 nt (150 nt for **d** and 200 nt for **h**) bin. Source data are provided as a Source Data file.

In eukaryotes, most mRNA decay is initiated by poly(A) tail deadenylation, including maternal mRNA decay during mammalian oocyte maturation^32^. It has been reported that deadenylation inhibition via *Btg4* depletion in mouse oocytes^18,19^ or *Btg4* mutation in human oocytes^20^ impedes maternal mRNA decay. Transcription has long been considered to be silent during oocyte maturation^33–36^. To further confirm this, we quantified PAIso-seq reads containing introns which is a good indication of new transcription, and found no obvious increase of reads with introns in in vitro matured oocytes compared to GV stage oocytes (Supplementary Fig. 3), indicating that the transcriptome we are looking are largely regulated by post-transcriptional regulation rather than new transcription. Therefore, we explored whether the impaired maternal mRNA decay seen in in vitro MII oocytes is due to defective RNA deadenylation. To test this, we analyzed the transcriptome-wide poly(A) tail length distribution in in vitro and in vivo MII oocytes and found a global accumulation of transcripts with 20–100 nt poly(A) tails in in vitro MII oocytes in both mice and humans (Fig. 2a, e). Note that the overall distribution of poly(A) tails shifts towards the short side in in vitro MII oocytes when normalized by total read counts (Supplementary Fig. 4a). However, although a bit counterintuitive, this does not mean that the poly(A) tails are deadenylated in in vitro MII oocytes, rather it indicates the deadenylation is defective. Our data suggest that many transcripts that normally undergoes full deadenylation retain a poly(A) tail of relatively short poly(A) tails (mainly between 20–100 nt, the transcripts between the magenta and the cyan curves in Fig. 2a) due to defective deadenylation when the MII oocytes were matured in vitro. The retained short poly(A) tails can be captured by PAIso-seq, while the fully deadenylated transcripts cannot. To further support this idea, we analyzed a published PAIso-seq data set of *Btg4* knockout MII oocytes^37^. Indeed, large amounts of transcripts with relatively short poly(A) tails accumulated in *Btg4* knockout MII oocytes (Supplementary Fig. 4b). Moreover, the relative total amount of poly(A)+ transcripts normalized by mitochondria coding transcript reads (3.6-fold in *Btg4* KO compared to WT) was very close to the fourfold poly(A)+ transcripts in *Btg4* KO compared to WT quantified by external spike-ins^19^, confirming the reliability of normalization by mitochondria coding transcripts. Similarly, the distribution of poly(A) tails shifts towards the short side in *Btg4* knockout MII oocytes when normalized by total read counts (Supplementary Fig. 4c). The compromised maternal mRNA deadenylation is typically more severe in transcripts from genes that are upregulated in in vitro MII oocytes (Figs. 1c, h, 2b, f), while transcripts from genes that are downregulated in in vitro MII oocytes showed an opposite pattern (Figs. 1c, h, 2c, g). For example, *Bmp15* transcripts with 20–100 nt poly(A) tails were significantly accumulated in in vitro MII oocytes of both mice and humans (Fig. 2d, h). These results demonstrate that the deadenylation of maternal mRNA is compromised in in vitro MII oocytes, which is conserved in both mice and humans.

**Fig. 3:**
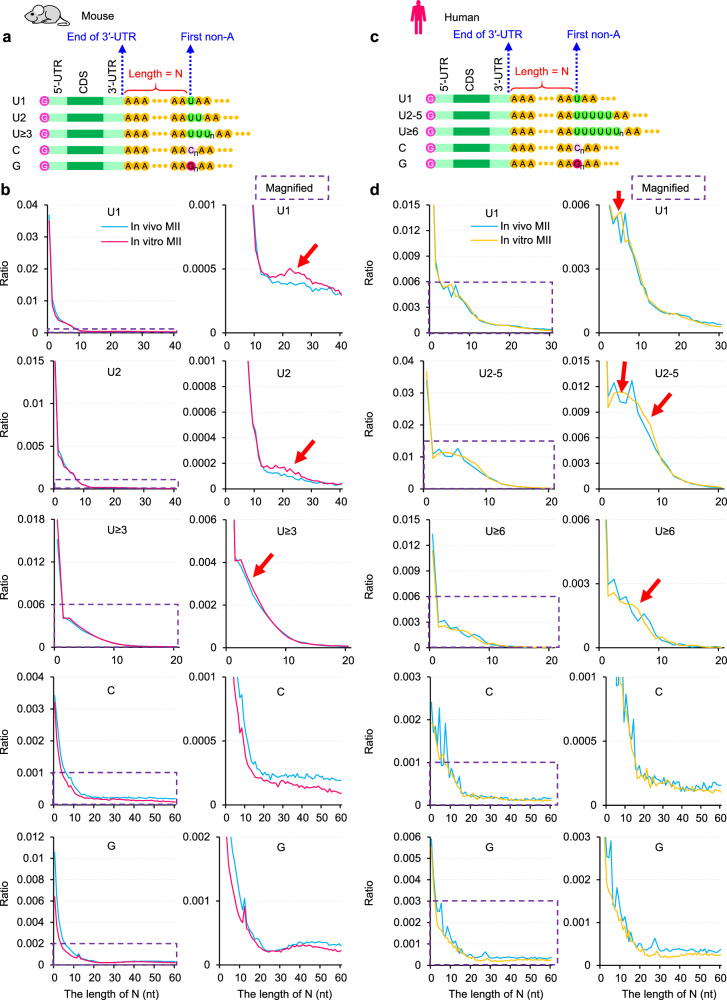
Mouse and human oocytes matured in vitro result in longer A residues before the first non-A. **a**, **c** Diagram depicting mRNA with internal non-A residues in mice (**a**) and humans (**c**). N represents the length of residues between the end of 3′ UTR and the first base of the longest consecutive U, C, or G residues in a poly(A) tail. **b** Histogram of the length of N and the ratio of U1, U2, U ≥ 3, C, and G residues (from top to bottom) in in vivo or in vitro MII oocytes in mice. **d** Histogram of the length of N and the ratio of U1, U2-5, U ≥ 6, C, and G residues (from top to bottom) in in vivo or in vitro MII oocytes in humans. Histograms (bin size = 1 nt) are normalized to the total number of transcripts with a poly(A) tail of at least 1 nt. Magnified views of the regions in the purple dotted squares are shown on the right. Red arrows highlight the proportion of transcripts with longer N increases in in vitro MII oocytes. Source data are provided as a Source Data file.

We report that non-A residues can be incorporated into maternal mRNA poly(A) tails through cytoplasmic polyadenylation followed by deadenylation during oocyte maturation, which is conversed in mice, rats, pigs, and humans^21–25^. When the deadenylation is impaired, the N (length between the end of 3′ UTR and the longest consecutive U, C, or G residues in poly(A) tails, Fig. 3a, c) is expected to be longer. This has been validated that the knockdown of *Btg4* in human zygotes and mouse MII oocytes results in longer N^21,23^. Therefore, we hypothesized that N should be longer in in vitro MII oocytes in which maternal mRNA deadenylation was impaired. As a result, we found that the N was longer for poly(A) tails with U residues in in vitro than in in vivo MII oocytes in both mice and humans (Fig. 3b, d). We also noticed that the ratio of poly(A) tails with C or G residues was lower in in vitro than in in vivo MII oocytes in mice and humans (Fig. 3b, d), reflecting lower incorporation of C and G residues. This suggests different mechanisms in U incorporation and C/G incorporation during oocyte maturation.

Altogether, these data demonstrate that impaired maternal mRNA decay is caused by impaired deadenylation in in vitro MII oocytes in both mice and humans.

### Impaired cytoplasmic polyadenylation of *Btg4* and *Cnot7* in in vitro MII oocytes

The compromised mRNA deadenylation in in vitro matured MII oocytes was very similar to the changes seen in *Btg4* knockout MII oocytes with a relatively smaller amplitude, suggesting that the deadenylation machinery might be dysregulated. Recent studies have demonstrated that CCR4-NOT deadenylase and its adapter BTG4 are critical for global maternal mRNA deadenylation during mouse and human OET^18–21,23^. Therefore, we examined whether the impaired deadenylation in in vitro MII oocytes was caused by problems that occurred with CCR4-NOT deadenylase and its adapter BTG4. The poly(A) tails of *Btg4* and *Cnot7* mRNA are short at the GV stage, and their translation is not initiated until cytoplasmic polyadenylation during oocyte maturation^18,19,38^. Therefore, we analyzed the transcript level and the poly(A) tail length of genes encoding components of the CCR4-NOT deadenylase and its adapter BTG4 in in vitro MII oocytes of mice and humans.

**Fig. 4:**
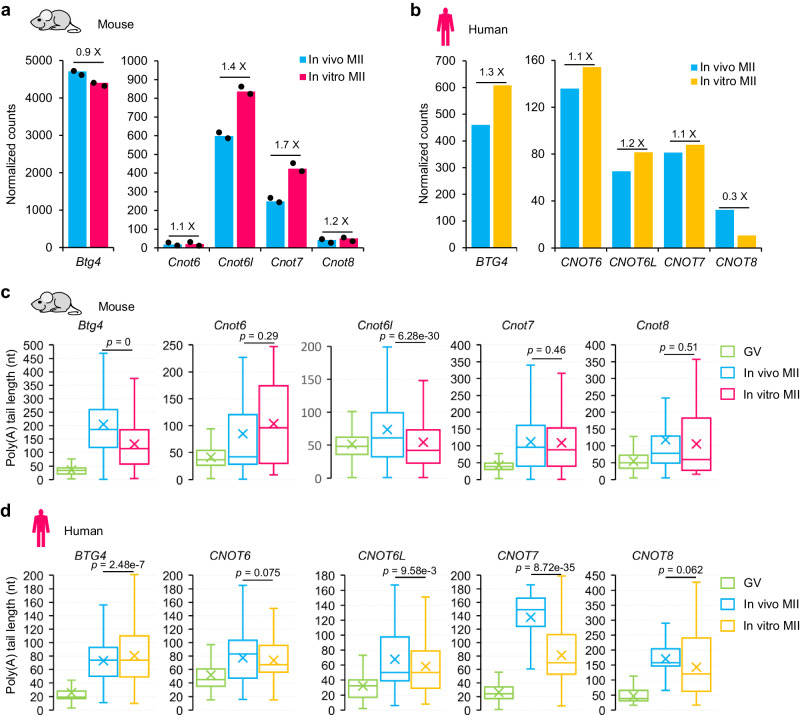
Polyadenylation of mRNAs encoding components of deadenylase complex is impaired in mouse and human oocytes matured in vitro. **a**, **b** Normalized counts of *Btg4* (left) and the four *Cnot* family (right) genes (*Cnot6*, *Cnot6l*, *Cnot7*, *Cnot8*) in the in vitro and in vivo MII oocytes for two biological replicates of mice (**a**) or combined data of humans (**b**). Individual data points are shown on the plots. Fold changes for each gene are shown on top of the column. **c**, **d** Box plot for the poly(A) tail length of *Btg4* and the four *Cnot* family genes in GV and MII oocytes matured in vivo or in vitro in mice (**c**) and humans (**d**). The *n* numbers for the box plots are 12900, 17360, 8618, 112, 63, 37, 1666, 2072, 1574, 1986, 871, 820, 933, 141, and 95 for (**c**) and 4326, 1164, 2887, 358, 343, 727, 412, 155, 386, 264, 197, 389, 184, 76, and 57 for (**d**) from left to right. For all the box plots, the “×” indicates the mean value, the central line represents the median value, the top and bottom of the box represent the value of the 25th and 75th percentile, and the boundary of the lower and the upper whiskers represent the minimum and the maximum value of the data set, respectively. The *p* values are derived from a two-tailed Student’s *t*-test. Source data are provided as a Source Data file.

At the transcript level, we found no or slight changes in the abundance of mRNA levels of these genes between in vitro and in vivo MII oocytes in mice and humans, except that *Cnot8* decreased dramatically in in vitro MII oocytes in humans (Fig. 4a, b). At the poly(A) tail length level, we observed a dramatic increase in poly(A) tail length for these genes during in vivo oocyte maturation in both mice and humans (Fig. 4c, d), reflecting the cytoplasmic polyadenylation of these genes. However, we found that the cytoplasmic polyadenylation of *Btg4* in in vitro MII oocytes in mice and *CNOT7* in in vitro MII oocytes in humans was strongly impaired (Fig. 4c, d), suggesting that these factors may not be translated at a normal level due to defective cytoplasmic polyadenylation.

**Fig. 5:**
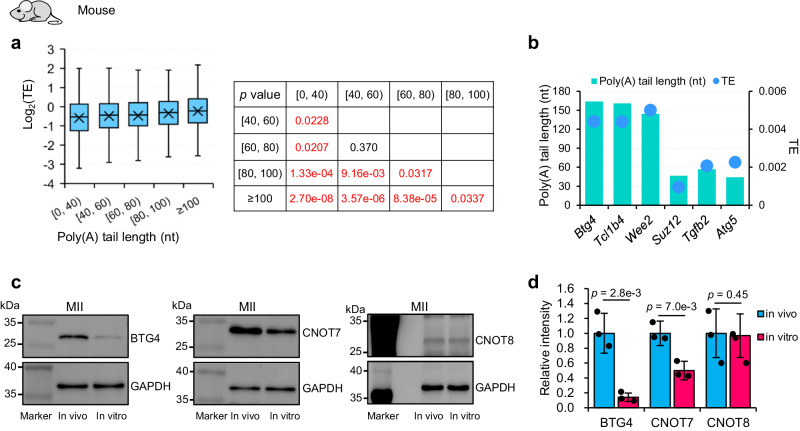
The protein levels of both Btg4 and Cnot7 are decreased in mouse oocytes matured in vitro. **a** Box plot of translational efficiency (TE, in log2 scale)^39^ of genes (*n* = 2951) grouped by the length of poly(A) tails in mouse in vivo MII oocytes (*n* number for the box plot from the left to right: 586, 908, 581, 431, and 445). The *p* values tested by two-tailed Student’s *t*-test between each of the two groups are shown on the right. **b** Examples of poly(A) tail length and TE^39^ for *Btg4*, *Tcl1b4*, *Wee2*, *Suz12*, *Tgfb2*, and *Atg5* in mouse in vivo MII oocytes. **c** Western blot analyses of the protein level of Btg4, Cnot7, and Cnot8 in mouse oocytes matured in vivo or in vitro. Fifty oocytes were used in each lane for the detection of Btg4 and Cnot7, while 200 oocytes were used in each lane for the detection of Cnot8. Blotting with anti-GAPDH was used as a control to confirm equal loading of the samples. **d** The signal intensities from three replicates of the protein level of Btg4, Cnot7, and Cnot8 were quantified and shown as bar plots. Error bars indicate the standard error of the mean (SEM) from three biological replicates. Individual data points are shown on the plots. The differences between oocytes matured in vivo and in vitro were statistically analyzed by one-tailed Student’s *t*-test with the *p* values shown on the top of the bars. For all the box plots, the “×” indicates the mean value, the central line represents the median value, the top and bottom of the box represent the value of the 25th and 75th percentile, and the boundary of the lower and the upper whiskers represent the minimum and the maximum value of the data set, respectively. Source data are provided as a Source Data file.

The poly(A) tail length of mRNA is known to be positively associated with translational efficiency (TE) in mouse GV to MI stage oocytes^24^. This positive association between poly(A) tail length and TE of mRNA has been suggested in mammalian MII oocytes but has never been examined transcriptome-widely. Therefore, we analyzed the relationship between our poly(A) tail length data and TE data from published polysome-seq in mouse MII oocytes^39^. We found that genes with longer poly(A) tails showed significantly higher TE in MII oocytes (Fig. 5a). For example, *Btg4*, *Tcl1b4*, and *Wee2* with poly(A) tail lengths around 140 nt showed much higher TE than *Suz12*, *Tgfb2*, and *Atg5* with poly(A) tail lengths around 50 nt (Fig. 5b). The genes encoding components for CCR4-NOT complex, including *Cnot6*, *Cnot6l*, *Cnot7*, and *Cnot8*, also showed good correlation between poly(A) tail length and TE (Supplementary Fig. 5), further suggesting the decreases of the poly(A) tails of *Btg4* and *Cnot7* might affect their translation in in vitro matured MII oocytes.

To experimentally confirm the defects in the BTG4-CCR4-NOT complex in oocytes matured in vitro, we performed a western blot to analyze the protein levels of its components, including Btg4, Cnot6, Cnot7, and Cnot8 using an equal number of mouse MII oocytes matured in vivo or in vitro. The results showed that both Btg4 and Cnot7 protein levels were decreased, though Cnot7 to a lesser extent (Fig. 5c, d). On the other hand, the protein level of Cnot8 does not show an obvious change between oocytes matured in vivo and in vitro (Fig. 5c, d). The Cnot6 antibody did not give a detectable signal, which impedes us to know the exact protein level of Cnot6 in oocytes matured in vivo or in vitro. Therefore, we can confidently conclude that protein levels of both Btg4 and Cnot7 are impaired in mouse oocytes matured in vitro.

Together, these results indicate that impaired deadenylation in in vitro MII oocytes is associated with the impaired cytoplasmic polyadenylation of mRNAs encoding CCR4-NOT deadenylase and its adapter Btg4, which leads to decreased protein levels of Btg4 and Cnot7.

## Discussion

**Fig. 6:**
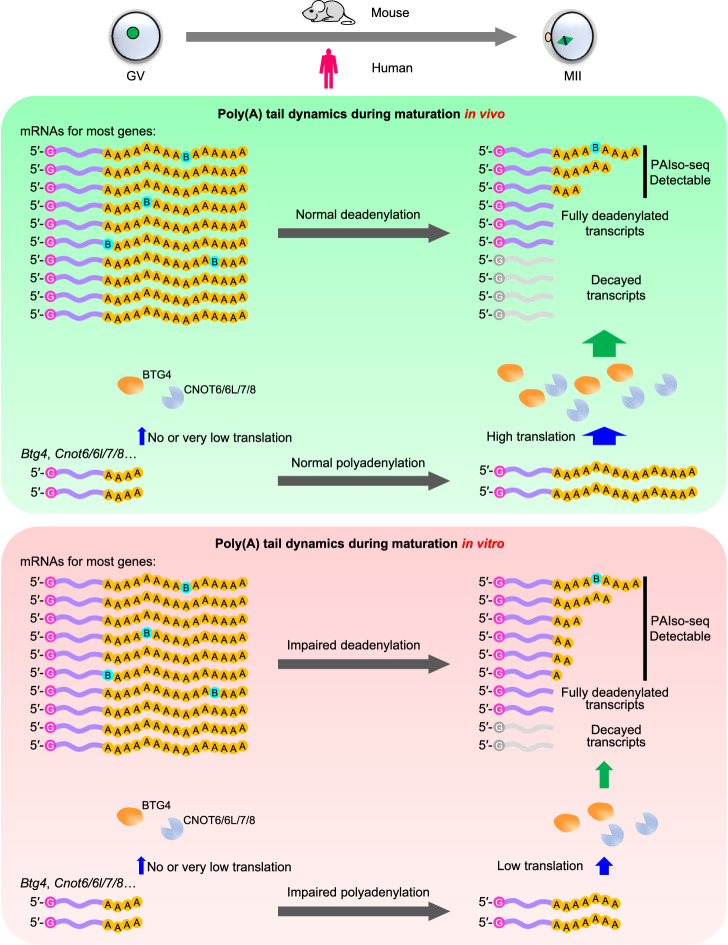
Summary of impaired maternal mRNA deadenylation during oocyte maturation in vitro. During oocyte maturation in vivo (top), the polyadenylation of mRNAs encoding components of deadenylase complex is normal, which results in high deadenylase abundance by high translation efficiency. Therefore, the global maternal mRNA deadenylation is normal. During oocyte maturation in vitro (bottom), the polyadenylation of mRNAs encoding components of deadenylase complex is impaired, which results in low deadenylase abundance by low translation efficiency. Therefore, the global maternal mRNA deadenylation is impaired. “B” in the tails indicates U, C, or G residue.

In vitro MII oocytes initially gained attention in ART for patients with PCOS^2^. More recently, it has been used in patients with repeated IVF failures^8^, resistant ovary syndrome, oocyte maturation problems^5^, and hormone-sensitive tumors^6^. Combining in vitro maturation and cryopreservation provides opportunities for women to postpone motherhood^5^. The reduced ability of in vitro MII oocytes to support embryo development has been reported, however, the molecular mechanism underlying its defect is still unclear. In this study, we revealed that the maternal mRNA decay mediated by deadenylation is defective in in vitro MII oocytes in both mice and humans, which is associated with the defective cytoplasmic polyadenylation of mRNAs encoding CCR4-NOT deadenylase and its adapter BTG4 (Fig. 6). We observed the accumulation of mRNA with short poly(A) tails rather than global extension of poly(A) tails in association with the decreased level of Btg4-CCR4-NOT complex. This is likely the common consequence of global changes of the CCR4-NOT complex activity as also evidenced by decreased the proportion of short poly(A) tails when LARP1, a negative regulator of the CCR4-NOT complex, is knocked down^40^. In addition, overcoming these defects will expand the available oocytes for assisted reproductive technology greatly that can help many patients.

The mechanism behind how the cytoplasmic polyadenylation of *Btg4* and *Cnot7* is impaired in in vitro MII oocytes is an interesting direction to pursue in the future. Previous studies reveal that translational activation of a subset of maternal mRNA is defective in denuded oocytes due to defective PI(3)K–AKT–mTOR pathway activation in oocytes in the absence of the surrounding somatic cells^41,42^. Therefore, it is likely that this can be one reason for impaired translational activation of *Btg4* and *Cnot7* and subsequent defective global deadenylation observed here in in vitro maturation in the absence of cumulus cells, which warrants further investigation in the future. It might also be interesting to investigate whether it is associated with other RNA modifications, such as m^6^A can associate with changes in poly(A) tail length^43^.

BTG4-mediated maternal mRNA decay is essential for successful OET in mice and humans, since *Btg4*-null oocytes were infertile due to developmental arrest at the cleavage stage^18–20^. In this study, we demonstrated that maternal mRNA deadenylation is impaired in mouse and human in vitro MII oocytes, which is associated with decreased developmental potential for supporting embryonic development. Improving the activity of CCR4-NOT deadenylase and its adapter BTG4 is likely to help overcome this problem, and we expect that these approaches, if successful, can one day promote the cytoplasmic maturation of oocytes under in vitro conditions. This will ultimately promote the future use of in vitro MII oocytes in human ART clinics.

Oocytes have recently been created in vitro from mouse pluripotent stem cells (PSCs), which can give rise to fertile and healthy offspring^31,44–47^. These groundbreaking results prove that it is possible to reproduce mice in the absence of natural oocytes. Using these methods in humans could provide hope for patients with infertility caused by oocyte abnormalities. Additionally, this procedure can contribute to regenerative medicine by providing oocytes for potential therapeutic cloning. Notably, PSC-derived MII oocytes also showed a higher expression level of many maternal mRNA than in vivo MII oocytes^31^. Therefore, our findings here can also be used to evaluate the quality of and improve PSC-derived oocytes, a subject of great potential that warrants further study.

## Methods

### Human oocytes

The use of human gametes for this research follows the Human Biomedical Research Ethics Guidelines (set by the National Health Commission of the People’s Republic of China in 2016), the 2016 Guidelines for Stem Cell Research and Clinical Translation (issued by the International Society for Stem Cell Research, ISSCR) and the Human Embryonic Stem Cell Research Ethics Guidelines (set by China National Center for Biotechnology Development on 24 December 2003). All the human-related experiments in this study are in compliance with these relevant ethical regulations. This study has received approval from the Institutional Review Board of Reproductive Medicine of Shandong University (201710) with respect to its aims and protocols. The human study was conducted in accordance with the criteria set by the Declaration of Helsinki. Immature germinal vesicle (GV) oocytes were donated by patients taking intracytoplasmic sperm injection (ICSI) treatments, and these immature oocytes were not used in regular clinical practice, because the patient already has enough oocytes for their own use. In vitro matured MII oocytes were from denuded GV oocytes that were kept in in vitro maturation (IVM) medium at 37 °C in an atmosphere with 5% CO_2_ for 23–27 h. The IVM medium consists of M199 medium (GIBCO, 11-150-059) with 20% Systemic Serum Substitute (Irvine Scientific, 99193) and 75 mIU/mL of recombinant follicle-stimulating hormone (Merck Serono). Three surplus MII oocytes were voluntarily donated by patients, who had successfully given birth and had excess frozen oocytes, for scientific research purposes with full informed consent. Before signing informed consent, patients have been informed in detail about the experimental objectives and destination of the donated samples. The sample donation is entirely voluntary, and no economic compensation has been provided. The donor women are 25–38 years old with tubal-factor infertility and their partners have healthy semen. Written informed consent was obtained from all oocyte donors. Single oocytes were used for PAIso-seq analysis.

### Mouse oocytes

CD1 (ICR) Mice were purchased from Beijing Vital River Laboratory Animal Technology Co., Ltd. and maintained in Individually Ventilated Cage (IVC) systems and specific pathogen-free (SPF) rooms (12/12 light-dark cycle, with temperatures maintained between 22−26 °C and humidity between 40−70%) according to the guidelines of the Animal Care and Use Committee of the Institute of Genetics and Developmental Biology, Chinese Academy of Sciences. GV oocytes were isolated from ovaries after injection with 10 U of pregnant mare serum gonadotropin (PMSG, Prospec, HOR-272). GV oocytes without cumulus cells were in vitro cultured in M16 medium (Sigma, MR-016) for 14 h to collect in vitro matured MII oocytes. To obtain MII oocytes, the mice were injected with 10 U of PMSG and 10 U of human chorionic gonadotropin (hCG, Prospec, HOR-250) at 46- to 48-h intervals. MII oocytes were isolated from the oviduct, without mating, 14 h after hCG injection.

### PAIso-seq library construction

The PAIso-seq libraries for human single oocytes (9 individual GV oocytes, four individual in vitro MII oocytes, and three individual in vivo MII oocytes) were constructed following the single-cell PAIso-seq protocol, and the PAIso-seq libraries for mouse oocytes (total RNA from around 400 oocytes for mouse GV, in vitro matured MII, and in vivo matured MII oocytes) were constructed following the bulk PAIso-seq protocol^26^, with the detailed protocol available^48,49^. A single human oocyte was washed with 1× phosphate-buffered saline (PBS, Invitrogen, AM9625) containing 0.1% bovine serum albumin (BSA, Sigma-Aldrich, A1933) three times, and transferred into a PCR tube containing 2.5 µl of cell lysis buffer (0.2% Triton X-100 (Sigma-Aldrich, T9284) containing 2 U/µL of RNase inhibitor (TaKaRa, 2313 A)) using a micro capillary pipette in the lowest possible volume (around 0.5 µl) to a final volume of around 3 µl. Then samples were incubated at 85 °C for 5 min for lysis and denaturation of the RNA, then put on ice immediately. Each sample was added with 1 µl of templated end extension oligo (50 µM, Supplementary Data 3) and 1.5 µl of nuclease-free water (Invitrogen, AM9938), incubated at 80 °C for 5 min followed by 37 °C for 10 min, and then put on ice immediately. Then each sample was incubated at 37 °C for 1 hour and then at 80 °C for 10 min after adding 4.5 µl of templated end extension mix (Final concentration: 1× SuperScript II first-strand buffer (Invitrogen, 18064-014), 5 mM DTT (Invitrogen, 18064-014), 0.5 mM each of dNTP mix (NEB, N0447L), 2 U/µl of RNase inhibitor (TaKaRa, 2313 A), and 0.5 U/µl of Klenow fragment 3′ → 5′ exo^−^ (NEB, M0212L)). After end extension, templated end extension oligos were digested by adding 1 µl USER enzyme (NEB, M5505L) and 15 µl nuclease-free water and then incubated at 37 °C for 30 min. Then, samples with different barcodes were mixed together into one tube and were purified with RNA Clean & Concentrator-5 kit (Zymo Research, R1016) in accordance with the manufacturer’s guidelines and were eluted with 7 µl nuclease-free water. For bulk samples, total RNA was added with 1 µl of templated end extension oligo (50 µM, Supplementary Data 3) and nuclease-free water to a total volume of 11 µl, incubated at 80 °C for 5 min followed by 37 °C for 10 min, and then put on ice immediately with the addition of 9 µl of templated end extension mix. Incubate the samples at 37 °C for 1 h and then at 80 °C for 10 min, followed by digestion with 1 µl USER enzyme and 30 µl nuclease-free water for 30 min at 37 °C. The samples were then purified with an RNA Clean & Concentrator-5 kit and were eluted with 7 µl nuclease-free water. After purification, the single-cell samples and the bulk samples were processed following the same procedures except different cycle number for PCR to achieve enough amount of cDNA. Each sample was added with 0.4 µl of RT primer (100 µM, Supplementary Data 3) and reversed transcribed with SuperScript II reverse transcriptase in the presence of 0.98 µM TSO (Supplementary Data 3), the sample was incubated at 42 °C for 90 min; 10 cycles of 50 °C for 2 min and 42 °C for 2 min; 70 °C for 15 min; and hold at 4 °C. Each sample was then PCR amplified by adding 25 µl of KAPA HiFi HotStart ReadyMix (2×, KAPA Biosystems, KK2601), 5 µl of IS PCR primer (10 µM, Supplementary Data 3), and water to a final volume of 50 µl. Preamplification was performed with the following program: 98 °C for 3 min; variable number of cycles of 98 °C for 20 s, 67 °C for 15 s, and 72 °C for 6 min; 72 °C for 10 min. Then the preamplification product was purified using 0.8× SPRIselect beads (Beckman Coulter, B23318) and eluted with 20 µl of nuclease-free water. 20 ng purified preamplification product was added with 400 µl of KAPA HiFi HotStart ReadyMix (2×), 80 µl of IS PCR primer (10 µM, Supplementary Data 3), and nuclease-free water to achieve an 800 µl mix, which was then split into 16× 50 μl tubes for large-scale PCR with the following program: 98 °C for 3 min; 10 cycles of 98 °C for 20 s, 67 °C for 15 s, 72 °C for 6 min; 72 °C for 10 min. Then, the large-scale PCR product was purified using 0.8× SPRIselect beads and eluted with 100 µl of nuclease-free water. Amplified cDNA products were size selected by Pure PB beads (PacBio, 100-265-900; 1x beads for cDNA with sizes above 200 bp and 0.4x beads for cDNA above 2 kb). These two parts of the sample were combined at equimolar concentrations for further library construction according to the standard PacBio Iso-Seq procedures and sequencing using PacBio Sequel I or Sequel II instruments under HiFi mode at Annoroad (a sequencing service provider in China, http://www.annoroad.com/).

### PAIso-seq sequencing data processing

The detailed protocol for PAIso-seq data processing follows our established pipelines^26,50^ and is available in Nature Protocols^48^. To demultiplex and extract the transcript sequence from the CCS reads, we first matched the barcodes in the CCS reads, and the reverse complements of the CCS reads, allowing a maximum of two mismatches or indels (insertions and deletions). CCS reads were oriented and split into multiple transcripts if multiple barcodes were matched. Then, to obtain the precise 3′ end position of the original RNA, we aligned the matched barcode to each transcript using the semi-global function “sg_dx_trace”, which did not penalize gaps at either the beginning or the end of query/barcode in parasail package^51^ and trimmed the barcode. Finally, the 3′-adapter and the 5′-adapter of each transcript were removed. Transcripts with a length greater than 50 nt were retained. Clean CCS reads were used for downstream analysis.

Clean CCS reads were aligned to the reference genome using minimap2 v.217-r941^52^ with parameters “-ax splice -uf --secondary=no -t 40 -L --MD --cs --junc-bed junction.bed”. The junction.bed file was converted from the reference gene annotation with “paftools.js gff2bed” in the minimap2 package. Read counts of each gene and gene assignments of each CCS reads were summarized by featureCounts v2.0.0^53^ with the parameters “-L -g gene_id -t exon -s 1 -R CORE -a reference.gtf” using the read alignments generated by minimap2. The clean CCS reads were then ready for downstream analysis.

### Poly(A) tail sequence extraction

The poly(A) tail sequence extraction follows our recently published procedures^23^. Clean CCS reads were aligned to reference genome using minimap2 (v.217-r941) with the following parameters “-ax splice -uf --secondary=no -t 40 -L --MD --cs --junc-bed junction.bed”^52^. Alignments with the “SA” (supplementary alignment) tag were ignored. The terminal clipped sequence of the CCS reads in the alignment bam file was used as the candidate poly(A) tail sequence. We defined a continuous score based on the transitions between the two adjacent nucleotide residues throughout the 3′-soft clip sequences. To calculate the continuous score, a transition from one residue to the same residue was scored as 0, and a transition from one residue to a different residue was scored as 1. The number of A, U, C, and G residues was also counted in the 3′-soft clip sequences of each alignment. The 3′-soft clip sequences with frequencies of U, C, and G, all greater or equal to 0.1 were marked as “HIGH_TCG” tails. The 3′-soft clips which were not marked as “HIGH_TCG” and with continuous scores less than or equal to 12 were considered poly(A) tails.

### Poly(A) tail length measurement

To accurately determine the lengths of poly(A) tails, we only quantified the poly(A) tail length from clean CCS reads with at least ten passes. The poly(A) tail length of a transcript was calculated as the length of the sequence, including U, C, or G residues if present. The poly(A) tail length of a gene was represented by the geometric mean of the poly(A) tail length of transcripts with tail length at least 1 nt from the given gene, because poly(A) tail length distribution of a gene follows a lognormal-like distribution^54,55^.

### Detection of non-A residues in poly(A) tails

To minimize errors introduced by the sequencer, we used clean CCS reads with at least ten passes to identify non-A residues in poly(A) tails. G, C, and U (presented as T in CCS reads) were counted in the poly(A) tail of each CCS read. The percentage of non-A transcripts (CCS reads which contained any non-adenosine residues) of a gene was calculated as the number of CCS reads containing at least one G, C, or U residue divided by the total number of CCS reads derived from the gene. Oligo-U (U ≥ 3 or U ≥ 6) refers to reads which contain at least three or six consecutive Us, mono-U refers to reads which contain single U but not any two consecutive Us, and U2 refers to reads which contain UU but not any three consecutive Us. U2-5 refers to reads which contain the longest 2 to 5 consecutive Us.

For assigning the positions of U, C, or G residues in poly(A) tails, a given poly(A) tail was first scanned for 3′-end U, C, or G residues, which if present were trimmed from the sequence, then searched for 5′-end U, C, or G residues, which were also trimmed, and finally searched for internal U, C, or G residues. As 3’-end non-A residues can not be accurately quantified by PAIso-seq, therefore, 3’-end non-A residues are not included in the analysis.

For calculating the N number for U, C, or G residues, a given poly(A) tail was first searched for the longest consecutive span of U, C, or G residues. The length of sequence before this longest consecutive stretch of U, C, or G residues was considered the N number. If a given poly(A) tail contained multiple stretches of longest consecutive U, C, or G residue, then the N number for this tail could not be determined, and thus, it was discarded from the N number analysis.

### Genome and gene annotation

The mouse genome sequence used in this study is from the following links. ftp://ftp.ensembl.org/pub/release-92/fasta/mus_musculus/dna/Mus_musculus.GRCm38.dna_rm.primary_assembly.fa.gz. The mouse genome annotation (including the nuclear-encoded mRNAs, lncRNAs and mitochondria encoded mRNAs) used in this study is from the following links.ftp://ftp.ensembl.org/pub/release-92/gtf/mus_musculus/Mus_musculus.GRCm38.92.gtf.gz.

The human genome sequence used in this study is from the following links. http://ftp.ebi.ac.uk/pub/databases/gencode/Gencode_human/release_36/GRCh38.primary_assembly.genome.fa.gz The human genome annotation (including the nuclear-encoded mRNAs, lncRNAs, and mitochondria encoded mRNAs) used in this study is from the following links.http://ftp.ebi.ac.uk/pub/databases/gencode/Gencode_human/release_36/gencode.v36.primary_assembly.annotation.gtf.gz.

### Western blot analysis of oocytes

An equal number (50 oocytes for detection of Btg4 and Cnot7, 200 oocytes for detection of Cnot8) of mouse oocytes matured in vivo or in vitro were lysed with 10 μl of 1×SDS loading buffer, incubated at 95 °C for 5 min and were immediately separated by SDS-PAGE. The samples were transferred onto a PVDF membrane (IPVH00010, Merck Millipore). The PVDF membrane was blocked and then incubated with primary antibodies (anti-Btg4, Abcam, ab206914; anti-Cnot7, Abcam, Ab195587; anti-Cnot8, Proteintech, 10752-1-AP; anti-Cnot6, Abcam, Ab221151; or anti-GAPDH, Proteintech, 60004-1-Ig; all these antibodies were used at a dilution of 1:1000) overnight at 4 °C. The membrane was washed five times with TBST, followed by incubation with suitable HRP-conjugated secondary antibody at 1:40,000 (anti-Rabbit IgG, Sigma, A6154-1ML; Anti-Mouse IgG, Sigma, A4416-.5 ML) for 1 h at room temperature. After washing with TBST five times, the signal was detected with SuperSignal West Femto maximum sensitivity substrate (34095, Thermo Scientific). The uncropped raw images are provided in the Source Data file.

### Reporting summary

Further information on research design is available in the Nature Portfolio Reporting Summary linked to this article.

### Supplementary information

Supplementary Information

Description of Additional Supplementary Files

Supplementary Data 1

Supplementary Data 2

Supplementary Data 3

Reporting Summary

### Source data

Source Data

## Data Availability

The PAIso-seq data for GV (the GV data has been described in our recent study^21^), in vitro MII, and in vivo MII oocytes in humans have been deposited in the Genome Sequence Archive for Human (GSA-Human) database hosted by the National Genomics Data Center under accession code HRA003115. The PAIso-seq data on GV, in vitro MII, and in vivo MII oocytes in mice have been described in our recent preprints^22–24^, and have been deposited in the Genome Sequence Archive (GSA) database hosted by National Genomics Data Center under accession code CRA008251. This study includes analysis of the following published data: Sha et al.^39^ in the Gene Expression Omnibus (GEO) database under accession code GSE118564, Xiong et al.^37^ in the GEO database under accession code GSE165782. Source data are provided with this paper.

## Code availability

Custom scripts used for PAIso-seq data analysis are available on github: https://github.com/Lulab-IGDB/polyA_analysis with a permanent reference to the version of the code in Zenodo^56^.
