# Supplementary material for: Maternal mRNA deadenylation is defective in in vitro matured mouse and human oocytes
Source: Nat Commun. 2024 Jul 2;15:5550. doi: 10.1038/s41467-024-49695-y (PMC11219934; doi:10.1038/s41467-024-49695-y)
Supplement: Supplementary file 1 — Supplementary Information [file 41467_2024_49695_MOESM1_ESM.pdf]

Maternal mRNA deadenylation is defective in in vitro matured mouse and human oocytes

Yusheng Liu[1,2,7] ✉, Wenrong Tao[3,7], Shuang Wu[2,4,7], Yiwei Zhang[2,4], Hu Nie[2,5], Zhenzhen Hou[3], Jingye Zhang[3], Zhen Yang[3], Zi-Jiang Chen[3,6], Jiaqiang Wang[4] ✉, Falong Lu[2,5] ✉ and Keliang Wu[3] ✉

[1]College of Life Science, Northeast Forestry University, Harbin, 150040, China.

[2]State Key Laboratory of Molecular Developmental Biology, Institute of Genetics and Developmental Biology, Innovative Academy of Seed Design, Chinese Academy of Sciences, Beijing 100101, China.

[3]Center for Reproductive Medicine, Shandong University, The Key laboratory of Reproductive Endocrinology, Ministry of Education, Shandong University, Jinan 250012, China.

[4]College of Life Science, Northeast Agricultural University, Harbin 150030, China.

[5]University of Chinese Academy of Sciences, Beijing 100049, China.

[6]Research Unit of Gametogenesis and Health of ART-Offspring, Chinese Academy of Medical Sciences (No.2021RU001), Jinan, Shandong, 250012, China.

[7]These authors contributed equally: Yusheng Liu, Wenrong Tao, Shuang Wu.

✉e-mail: liuys1126@foxmail.com; wangjiaqiang@neau.edu.cn; fllu@genetics.ac.cn; wukeliang_527@163.com.

Supplemental Information

The supplementary information consists of 5 Supplementary Figures.

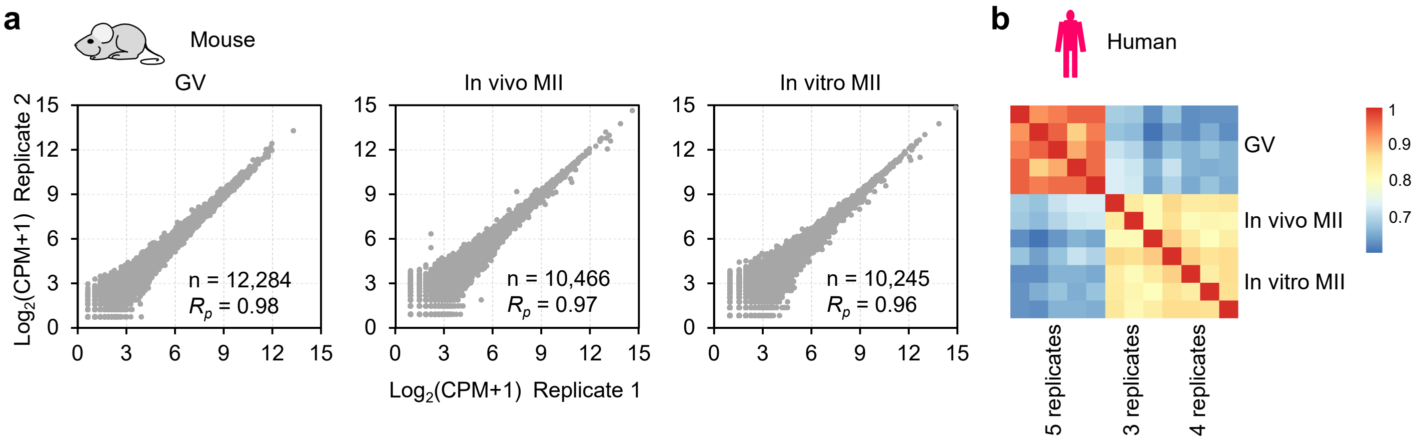

Supplementary Fig. 1 | Oocyte PAIso-seq datasets show good reproducibility.

a, Gene expression correlation between two replicates for mouse GV, in vivo matured MII and in vitro matured MII oocytes measured by PAIso-seq. Pearson's correlation coefficient (*Rp*) and number of genes are shown on the bottom right of each plot.

b, Gene expression correlation (Pearson's correlation coefficient) between each single human GV, in vivo matured MII and in vitro matured MII oocytes measured by PAIso-seq. CPM: counts per million. Source data are provided as a Source Data file.

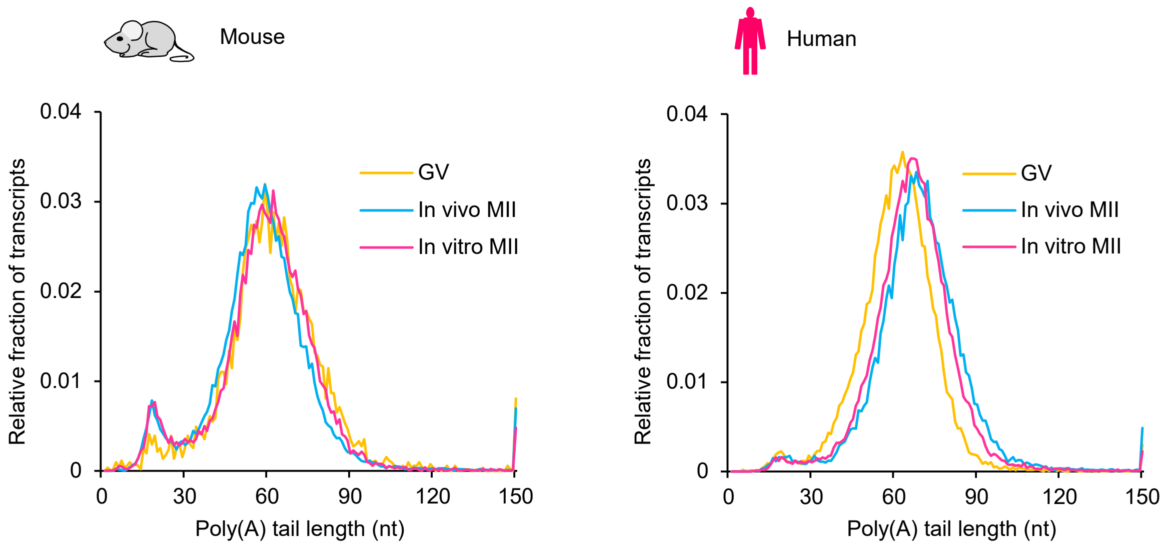

Supplementary Fig. 2 | The poly(A) tails of mitochondrial genome-encoded polyadenylated mRNAs are minimally affected during oocyte maturation.

Histogram of poly(A) tail length for all MT-mRNA transcripts in GV, in vivo matured MII and in vitro matured MII oocytes measured by PAIso-seq in mice (left) and in humans (right). Transcripts with poly(A) tail length of 150 nt or longer are combined in the 150 nt data point. Source data are provided as a Source Data file.

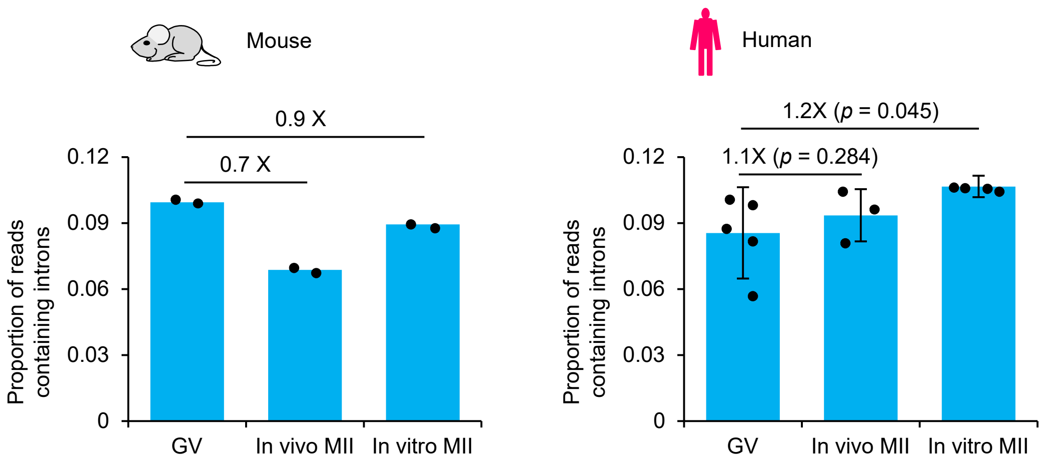

Supplementary Fig. 3 | No obvious transcription taken place in in vitro matured oocytes. Proportion of reads containing introns in GV, in vivo matured, and in vitro matured oocytes in mice and in humans. Individual data points are shown on the plots. Error bars indicate the standard error of the mean (SEM) from independent single oocytes (GV, n = 5; in vivo MII, n = 3; in vitro MII, n = 4) analyzed in human samples. The *p*-values tested by one-tailed Student's *t*-test between the GV and MII oocytes are shown on top of the plots. Source data are provided as a Source Data file.

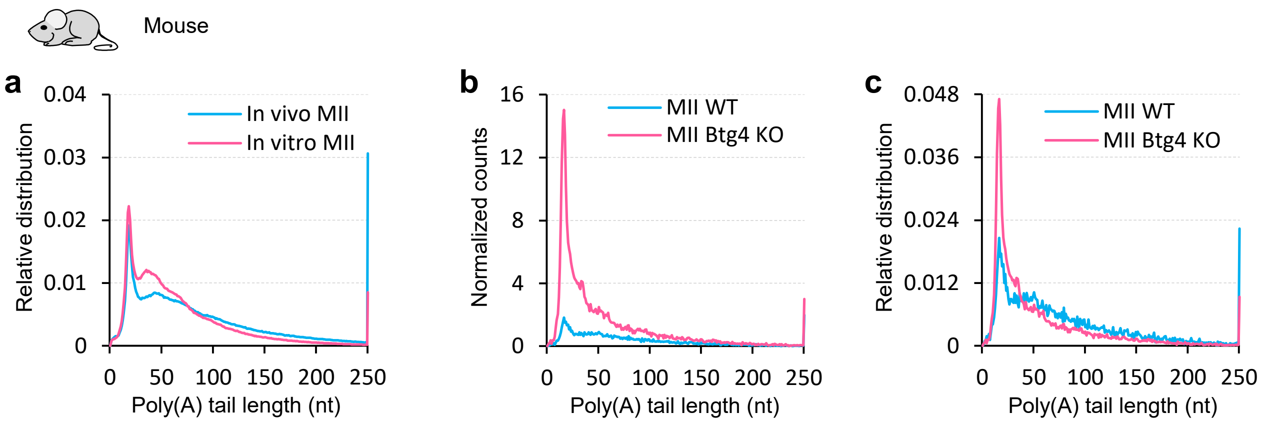

Supplementary Fig. 4 | Transcript-level poly(A) tail length in MII oocytes in mice.

The distribution (histogram, bin size = 1 nt) of the poly(A) tail lengths for each transcript of annotated coding genes in in vivo matured and in vitro matured MII oocytes normalized by total read counts (**a**), as well in Btg4 knockout MII oocytes normalized by counts of reads mapped to protein-coding genes in the mitochondria genome (**b**) or total read counts (**c**). Transcripts with poly(A) tails of at least 1 nt are included in the analysis. Transcripts with poly(A) tail lengths greater than 250 nt are included in the 250 nt bin. Raw data for Btg4 knockout MII oocytes were downloaded from a recent publication[37]. Source data are provided as a Source Data file.

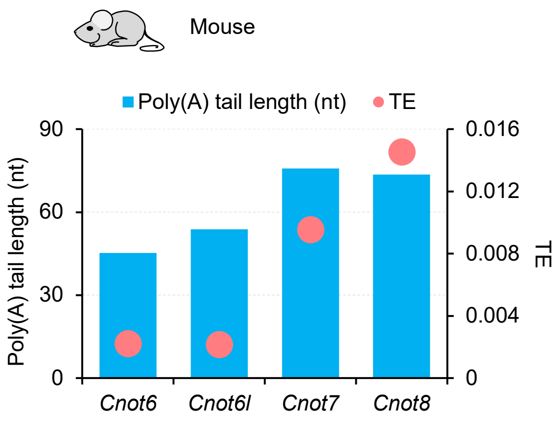

Supplementary Fig. 5 | Longer poly(A) tail associates with higher TE.

Examples of poly(A) tail length and TE for *Cnot6*, *Cnot6l*, *Cnot7* and *Cnot8*. Source data are provided as a Source Data file.
