# Supplementary material for: Maternal mRNA deadenylation is defective in in vitro matured mouse and human oocytes
Source: Nat Commun. 2024 Jul 2;15:5550. doi: 10.1038/s41467-024-49695-y (PMC11219934; doi:10.1038/s41467-024-49695-y)
Supplement: Supplementary file 2 — Description of Additional Supplementary Files [file 41467_2024_49695_MOESM2_ESM.pdf]

Description of Additional Supplementary Files

File Name: Supplementary Data 1

Description: Normalized gene expression level in mouse MII oocytes measured by PAIso-seq (normalized CPM). The p-value or FDR for differential gene expression between in vivo and in vitro MII oocytes were included.

File Name: Supplementary Data 2

Description: Normalized gene expression level in human MII oocytes matured in vitro and in vivo measured by PAIso-seq (normalized CPM).

File Name: Supplementary Data 3

Description: Oligos used for PAIso-seq in this study and the number of CCS reads for each of the sample.
