# Supplementary material for: Maternal mRNA deadenylation is defective in in vitro matured mouse and human oocytes
Source: Nat Commun. 2024 Jul 2;15:5550. doi: 10.1038/s41467-024-49695-y (PMC11219934; doi:10.1038/s41467-024-49695-y)
Supplement: Supplementary file 6 — Reporting Summary [file 41467_2024_49695_MOESM6_ESM.pdf]

nature portfolio

Corresponding author(s): Falong Lu

Last updated by author(s): May 26, 2024

Reporting Summary

Nature Portfolio wishes to improve the reproducibility of the work that we publish. This form provides structure for consistency and transparency in reporting. For further information on Nature Portfolio policies, see our Editorial Policies and the Editorial Policy Checklist.

Statistics

For all statistical analyses, confirm that the following items are present in the figure legend, table legend, main text, or Methods section.

n/a	Confirmed	
☐	☒	The exact sample size (*n*) for each experimental group/condition, given as a discrete number and unit of measurement
☒	☐	A statement on whether measurements were taken from distinct samples or whether the same sample was measured repeatedly
☐	☒	The statistical test(s) used AND whether they are one- or two-sided<br>*Only common tests should be described solely by name; describe more complex techniques in the Methods section.*
☒	☐	A description of all covariates tested
☒	☐	A description of any assumptions or corrections, such as tests of normality and adjustment for multiple comparisons
☐	☒	A full description of the statistical parameters including central tendency (e.g. means) or other basic estimates (e.g. regression coefficient) AND variation (e.g. standard deviation) or associated estimates of uncertainty (e.g. confidence intervals)
☐	☒	For null hypothesis testing, the test statistic (e.g. *F*, *t*, *r*) with confidence intervals, effect sizes, degrees of freedom and *P* value noted<br>*Give P values as exact values whenever suitable.*
☒	☐	For Bayesian analysis, information on the choice of priors and Markov chain Monte Carlo settings
☒	☐	For hierarchical and complex designs, identification of the appropriate level for tests and full reporting of outcomes
☐	☒	Estimates of effect sizes (e.g. Cohen's *d*, Pearson's *r*), indicating how they were calculated

Our web collection on statistics for biologists contains articles on many of the points above.

Software and code

Policy information about availability of computer code

Data collection	The sequencing data was acquired from Pacific BioSciences (PacBio) sequencing platform sequel I or sequel II HiFi mode with the default parameters.
Data analysis	Ccs (version 5.0.0) was used to process subreads generated by PacBio SMRTbell sequencing. Minimap2 (v.217-r941) was used to align ccs reads to reference genome. Read counts of each gene and gene assignments of each CCS reads were summarized by featureCounts v2.0.0. Custom scripts used for PAIso-seq data analysis are available in github: https://github.com/Lulab-IGDB/polyA_analysis with a permanent reference to the version of the code in Zenodo (https://doi.org/10.5281/zenodo.11262468).

For manuscripts utilizing custom algorithms or software that are central to the research but not yet described in published literature, software must be made available to editors and reviewers. We strongly encourage code deposition in a community repository (e.g. GitHub). See the Nature Portfolio guidelines for submitting code & software for further information.

Data

Policy information about availability of data

All manuscripts must include a data availability statement. This statement should provide the following information, where applicable:

- Accession codes, unique identifiers, or web links for publicly available datasets
- A description of any restrictions on data availability
- For clinical datasets or third party data, please ensure that the statement adheres to our policy

The PAIso-seq data for GV (the GV data has been described in our recent study), in vitro MII, and in vivo MII oocytes in humans have been deposited in the Genome Sequence Archive for Human (GSA-Human) database hosted by National Genomics Data Center under accession code HRA003115 [https://ngdc.cncb.ac.cn/gsa-human/browse/HRA003115]. The PAIso-seq data on GV, in vitro MII, and in vivo MII oocytes in mice have been described in our recent preprints, and have been deposited in the Genome Sequence Archive (GSA) database hosted by National Genomics Data Center under accession code CRA008251 [https://ngdc.cncb.ac.cn/gsa/browse/CRA008251]. This study includes analysis of the following published data: Sha et al. in the Gene Expression Omnibus (GEO) database under accession code GSE118564 [https://www.ncbi.nlm.nih.gov/geo/query/acc.cgi?acc=GSE118564], Xiong et al. in the GEO database under accession code GSE165782 [https://www.ncbi.nlm.nih.gov/geo/query/acc.cgi?acc=GSE165782]. Source data are provided with this paper.

Research involving human participants, their data, or biological material

Policy information about studies with human participants or human data. See also policy information about sex, gender (identity/presentation), and sexual orientation and race, ethnicity and racism.

Reporting on sex and gender	In this experiment, the research object is the oocytes and does not involve sex and/or gender. Therefore, the sex/gender information is not applicable to this study.
Reporting on race, ethnicity, or other socially relevant groupings	Not available.
Population characteristics	The donor women are 25–38 years old with tubal-factor infertility and their partners have healthy semen.
Recruitment	Patients with a large number of follicles are communicated clearly about the research purpose in advance before oocyte retrieval to see if they are willing to donate immature oocytes for scientific research with no compensation, and also be ensured that the donated oocytes will be used for research only but not any clinical purposes. Written informed consent is signed by all the donors. When obviously immature GV or MI oocytes are identified by an embryologist during oocyte denuding, another embryologist will confirm the oocyte maturity and then check whether the patient has signed the informed consent for donation. The oocytes meeting the requirements will be collected for subsequent scientific research. Three surplus MII oocytes were voluntarily donated by patients, who had successfully given birth and had excess frozen oocytes, for scientific research purposes with full informed consent.
Ethics oversight	Institutional Review Board of Reproductive Medicine of Shandong University

Note that full information on the approval of the study protocol must also be provided in the manuscript.

Field-specific reporting

Please select the one below that is the best fit for your research. If you are not sure, read the appropriate sections before making your selection.

☒ Life sciences ☐ Behavioural & social sciences ☐ Ecological, evolutionary & environmental sciences

For a reference copy of the document with all sections, see nature.com/documents/nr-reporting-summary-flat.pdf

Life sciences study design

All studies must disclose on these points even when the disclosure is negative.

Sample size	We chose sample size based on literatures in the field and experimental knowledge.
Data exclusions	No data were excluded from the analyses.
Replication	Two biological replicates were performed for each mouse PAIso-seq experiments. Single oocytes (9 individual GV oocytes, 4 individual in vitro MII oocytes, and 3 individual in vivo MII oocytes) were conducted for human PAIso-seq experiments. All replicates were successful.
Randomization	Samples were randomly collected into groups.
Blinding	Analysis was objective and did not require blinding.

Reporting for specific materials, systems and methods

We require information from authors about some types of materials, experimental systems and methods used in many studies. Here, indicate whether each material, system or method listed is relevant to your study. If you are not sure if a list item applies to your research, read the appropriate section before selecting a response.

Materials & experimental systems

n/a	Involved in the study	
☐	☒	Antibodies
☒	☐	Eukaryotic cell lines
☒	☐	Palaeontology and archaeology
☐	☒	Animals and other organisms
☒	☐	Clinical data
☒	☐	Dual use research of concern
☒	☐	Plants

Methods

n/a	Involved in the study	
☒	☐	ChIP-seq
☒	☐	Flow cytometry
☒	☐	MRI-based neuroimaging

Antibodies

Antibodies used	anti-Btg4, Abcam, ab206914; anti-Cnot7, Abcam, Ab195587; anti-Cnot8, Proteintech, 10752-1-AP; anti-Cnot6, Abcam, Ab221151; or anti-GAPDH, Proteintech, 60004-1-Ig; all these antibodies were used at a dilution of 1:1000 for Western blot.
Validation	The antibodies has been validated for the species used here on the manufacturer's website. And we confirmed the size of the western blot band matches the expected size.

Animals and other research organisms

Policy information about studies involving animals; ARRIVE guidelines recommended for reporting animal research, and Sex and Gender in Research

Laboratory animals	CD1 (ICR) mice were purchased from Beijing Vital River Laboratory Animal Technology Co., Ltd and bred in our facility in Individually Ventilated Cage (IVC) systems and specific pathogen-free (SPF) rooms (12/12 light-dark cycle, with temperatures maintained between 22 - 26°C and humidity between 40 - 70%) . Male and female mice between the age group of 7-8 weeks were used in this study.
Wild animals	The study did not involve wild animals.
Reporting on sex	This study focus on oocytes which are only available for females but not males.
Field-collected samples	This study did not involve field samples.
Ethics oversight	Mice were maintained in compliance with the guidelines of the Animal Care and Use Committee of the Institute of Genetics and Developmental Biology, Chinese Academy of Sciences (CAS).

Note that full information on the approval of the study protocol must also be provided in the manuscript.

Plants

Seed stocks	Not available.
Novel plant genotypes	Not available.
Authentication	Not available.
